# Modified Starch as a Filter Controller in Water-Based Drilling Fluids

**DOI:** 10.3390/ma13122794

**Published:** 2020-06-20

**Authors:** Diana Soto, Orietta León, José Urdaneta, Alexandra Muñoz-Bonilla, Marta Fernández-García

**Affiliations:** 1 Laboratorio de Polímeros y Reacciones, Escuela de Ingeniería Química, Facultad de Ingeniería, Universidad del Zulia, Sector Grano de Oro, Maracaibo 4011, Venezuela; dsoto@fing.luz.edu.ve (D.S.); joseurdaneta1@hotmail.com (J.U.); 2 Departamento de Química y Propiedades de Materiales Poliméricos, Instituto de Ciencia y Tecnología de Polímeros (ICTP-CSIC), C/Juan de la Cierva 3, 28006 Madrid, Spain; sbonilla@ictp.csic.es; 3 Interdisciplinary Platform for Sustainable Plastics towards a Circular Economy-Spanish National Research Council (SusPlast-CSIC), 28006 Madrid, Spain

**Keywords:** starch, itaconic acid, graft copolymer, drilling fluids

## Abstract

Herein, the effectiveness of an itaconic acid (IA) graft copolymer on native corn starch (NCS) as a filter control agent in fresh water-based drilling fluids (WBDFs) was evaluated. The copolymer (S-*g*-IA_APS) was synthesized by conventional radical dispersion polymerization using the redox initiation system (NH_4_)_2_S_2_O_8_/NaHSO_3_. The modification of the starches was verified by volumetry, Fourier transform infrared spectroscopy (FTIR), thermogravimetric analysis (TGA), and scanning electron microscopy (SEM). Then, three WBDFs were formulated in which only the added polymer (NCS, S-*g*-IA_APS, and a commercial starch (CPS)) was varied to control the fluid losses. The physico-chemical, rheological, and filtering properties of the formulated systems were evaluated in terms of density (ρ), pH, plastic viscosity (µ_p_), apparent viscosity (µ_a_), yield point (Y_p_), gel strength (R_g_), and filtrated volume (V_API_). In order to evaluate the resistance to temperature and contaminants of the WBDFs, they were subjected to high pressure and high temperature filtering (V_HPHT_). The filter control agents were also subjected to aging and contamination with cement and salt. The S-*g*-IA_APS addition improved the filtering behavior at a high pressure and temperature by 38%.

## 1. Introduction

The use of drilling muds is an inseparable part of the rotary drilling process [1]. Drilling fluids or muds consist of suspensions of clay particles in water or oil [2]. When drilling an oil well, the mud circulates down through the drilling pipe, then up through the annular space between the drilling pipe and the drilling hole, at a high speed [3,4]. Although the factors that guide the selection of a base fluid and mud additives are not simple, both technical and environmental factors should be taken into account [5,6]. In response to environmental requirements, most drilling muds currently used in oil fields are formulated in water. The additives used are intended to ensure that the sludge meets some functional demands of the fluid, such as rheology, density, reactivity, fluid loss control property, etc. [1,5]. These features are a prerequisite for the use of mud in drilling operations to perform specific functions, such as cooling and lubricating the drill bit, bringing drill cuttings (debris) to the surface, reducing friction, as well as forming a fine filter cake (plaster) on the borehole walls [3,7,8]. Plaster formation minimizes fluid loss, which is due to a high hydrostatic pressure compared to a formation pressure, through high permeability rock formations [6]. A proper filtration control is very important to avoid some problems of drilling such as excessive torque, draw-well instability, and formation damage.

For the last fifty years, starch has been used extensively in its native or modified forms, as a filtration control agent in drilling muds, due to its abundance, low cost, and biodegradability [4,8,9,10]. Regarding modified starches, pregelatinized, oxidized, etherified, and grafted starches have been used [4,5,11,12]. These modifications have been made with the main objective of improving the water solubility and thermal stability of the starch, as well as its resistance to bacterial attack and consequently improving fluid loss properties [4]. Of all the modifications, grafting starch with vinyl monomers seems to be the most effective method to introduce different functional groups into the polysaccharide and increase its molecular mass, thus achieving a tailored macromolecular architecture and performance as stated by Guo and Peng, Athawale and Lele, Fares et al., Soto et al. and Singh et al. [4,13,14,15,16]. However, very scarce research has been reported on the application of grafted copolymers as filtration control agents to WBDFs, possibly due to trademade policies.

In spite of the abovementioned, grafting of synthetic monomers into the starch backbone has been reported to lead to materials having combined rheological behaviors of both natural and synthetic polymers. Moreover, when graft copolymers having longer chains are added to an aqueous dispersion of clay particles, adsorption of the copolymer takes place on the surface thereof, leading to the formation of a polymeric layer at the equilibrium. The natural skeleton (less soluble in water) is in direct contact with the clay surface, while the grafted side chains (more soluble in water) expand in the solution to form brushes that stabilize the aqueous clay dispersion [4,5].

The use of itaconic acid (IA) graft copolymers on corn starch as filter controllers in WBDFs has not been described so far. An itaconic acid is nowadays obtained from renewable resources by fermentation but previously was obtained by the distillation of a citric acid [17]. This monomer is biodegradable, economic, and very interesting to produce polyelectrolytes, since they contain two ionizable groups with two different values of ionization constants (K_a_), which can form hydrogen bonds. Having this in mind, this work presents the preparation of an IA grafted corn starch and its use as a filter controller. The physico-chemical and rheological properties of the prepared materials were evaluated and compared with native and commercial starches used in the sludges.

## 2. Materials and Methods

### 2.1. Materials

Native corn starch (NCS) was provided by Alfonso Rivas & Cía (Caracas, Venezuela). The hydrochloric acid (HCl, 37%), sodium hydroxide (NaOH, 99%), itaconic acid (IA, ≤99%), and baryte (Barium sulphate, 99%) were provided from Merck (Darmstadt, Alemania). Hydroxylamine hydrochloride (NH_2_OH.HCl, 99%) was provided from Lobal Chemie (Mumbai, India); chloride sodium (NaCl, 99.8%) from Baker (Madrid, Spain); ammonium persulfate ((NH_4_)_2_S_2_O_8_, 98%), bentonite (99.9%), and commercial potato starch (CPS, ≤99.6%) from Sigma-Aldrich (St. Louis, Kansas, MO, USA); and sodium bisulfite (NaHSO_3_, 66.9%) from Mallinckrodt (Staines, Reino Unido). All materials were used as received.

### 2.2. Copolymerization of Corn Starch with an Itaconic Acid, S-g-IA_APS

The preparation of a S-*g*-IA_APS copolymer was performed following the procedure previously described [15]. Briefly, an aqueous dispersion of NCS with a concentration of 0.6173 M (5 g of starch in 50 mL of distilled water) of anhydroglucose units (AGU) was prepared. The dispersion was filled into a three neck flask and heated at 75 °C for 15 min with gentle stirring. To this dispersion, 100 mL of water was added and then, it was cooled to 60 °C. Next, 8 × 10^−3^ M of the (NH_4_)_2_S_2_O_8_ initiator and 3 × 10^−2^ M of the NaHSO_3_ activator solutions were added to the polysaccharide; and the suspension was kept in contact with the redox pair for 10 min. After this preoxidation step, 0.185 M of the non-neutralized IA monomer, previously dissolved in a volume of 90 mL of distilled water, was added. Immediately, the final volume was increased to 250 mL with distilled water and allowed to react for 3 h. Then, the mixture was cooled down and precipitated in ethanol to obtain the modified starch. The precipitate was dried at 40 °C until a constant weight.

### 2.3. Starch Characterization 

#### 2.3.1. Determination of Carbonyl, –C(O)H, and Carboxyl, –COOH, Groups Contents

The determination of the amount of these groups in the different starches was made following previous procedures [15,18,19]. The percentage of groups –C(O)H was obtained through Equation (1) and the percentage of –COOH groups by Equation (2).

(1) $$–C\left(O\right)H\;\left(\%\right)=\frac{{(V}_{B}-V_{M}{)\;\times \;C}_{\mathrm{HCl}}\;\times \;0.28\;\times \;100}{W_{D}}$$

(2) $$–\mathrm{COOH}(\%)=\frac{{(V}_{M}-V_{B}{)\;\times \;C}_{\mathrm{NaOH}}\;\times \;0.045\;\times \;100}{W_{D}}$$

where V_B_ and V_M_ are the titrated volumes in mL consumed by the blank and the starch sample in the respective assay, C_HCl_ is the concentration of HCl in mol/L, C_NaOH_ is the concentration of NaOH in mol/L, and W_D_ is the mass of the dry sample in g. Briefly, the –C(O)H content is determined as follows: The starch (0.4 g) was dispersed in 10 mL of water, transferred to a 50 mL beaker, and heated to 100 °C for gelatinization; then it was slowly cooled to 40 °C. The pH of the solution was adjusted to 3.2 with 0.1 M HCl and then, 1.5 mL of hydroxylamine hydrochloride was added. Then, the mixture was allowed to react at this temperature for 4 h, the excess hydroxylamine hydrochloride was finally titrated with 0.1 M HCl to pH = 3.2. A blank containing only hydroxylamine hydrochloride was titrated in the same way. In the case of –COOH, its content is determined as follows: 2.5 mL of HCI 0.1 N were added to 0.2 g of starch, and the mixture was allowed to rest for 30 min with intermittent stirring. The slurry was filtered through a Whatman No.1 qualitative filter paper (Grade 4) and washed with distilled water until it was free from chlorine. Then, the starch was transferred into a 50 mL beaker, and 30 mL of distilled water was added. Afterwards, the mixture was refluxed during 15 min for the complete gelatinization of the starch; then, it was slowly cooled to room temperature followed by titration with a NaOH 0.01 N solution with phenolphthalein as an indicator. A blank test was also performed. All the samples were tested at least in triplicate.

#### 2.3.2. Fourier Transform Infrared Spectroscopy (FTIR)

The infrared spectra of the different dried starches were recorded with a FTIR spectrophotometer (Spectrum One, Perkin Elmer/PIKE MIRacle™ Technologies, Wellesley, MA, USA) measured in the ATR mode and with a spectral resolution of 0.5 cm^−1^.

#### 2.3.3. Thermogravimetric Analysis (TGA)

The thermal degradation study was performed on a TGA Q500 (TA Instruments, New Castle, DE, USA). The instrument was calibrated in the temperature and mass by standard methods. The dynamic experiments were performed in nitrogen at a heating rate of 10 °C/min in the temperature range between 40 and 800 °C. The average sample size was approximately 5 mg and the nitrogen flow rate was 50 cm^3^/min. The activation energy (E_a_) of the decomposition of the samples was calculated by the Broido method [20] as follows:

(3) $$\ln\;\left[\ln(\frac{1}{Y})\right]=-\frac{E_{a}}{R}\times \frac{1}{T}+\mathrm{constant}$$

where E_a_ is the activation energy in kJ/mol, R is the ideal gas constant, T is the temperature in K, and Y is the unconverted mass fraction, which is obtained from Equation (4):

(4) $$Y=\frac{w_{t}{-w}_{\infty }}{w_{0}{-w}_{\infty }}$$

where w_t_ is the mass of the sample at a temperature T, w_0_ is the initial mass of the sample, and w_∞_ is the mass of ash recorded at the maximum temperature of the analysis.

#### 2.3.4. Scanning Electron Microscopy (SEM)

The morphology and the particle size were examined by the scanning electron microscopy using a Philips XL30 microscope (Philips, Mahwah, NJ, USA) with an acceleration voltage of 25 kV. The samples were coated with 5 nm of gold/palladium (80/20) prior to visualization. SEM images to obtain particle sizes and particle size distributions were processed and quantified using the Fiji distribution of ImageJ software.

### 2.4. Preparation of Water-Based Drilling Fluids

Table 1 lists the components and amounts used in the formulation of the WBDFs, along with a general description of their function. The design and composition of the fluid to be developed depends on the physical-chemical characteristics of the ground to be drilled, final depth, availability, cost, and care for the environment. However, in conventional drilling fluids the main additives are viscosifiers, densifiers, and filter controllers. In this work, a base fluid (F1) without a filter controller was first prepared. Then, three WBDFs were prepared (F2, F3, and F4), in which only the starch used as a filter controller was varied (NCS, S-*g*-IA_APS, and CPS (Sigma-Aldrich number S2004), respectively). Bentonite is usually added in an amount between 10.5 and 31.5 g per 350 mL, according to the desired rheological properties, while the typical concentration of NaOH varies from 0.20 to 4 g per 350 mL [7]. Most of the filter controllers used in WBDFs achieve their effectiveness at a concentration between 0.5 and 8 g per 350 mL. Baryte is the most used densifier; its quantity in the formulation was determined by a mass balance to obtain a fluid with a theoretical density of 1.50 g/mL.

For the preparation of each fluid, 350 mL of fresh water were measured. The amount of water was poured into a stainless steel beaker and stirred in a sludge mixer. Then, the bentonite was slowly added under vigorous stirring for 20 min to allow its dispersion and swelling. Subsequently, the starch used as a filter controller was added, except in F1, with vigorous stirring, avoiding as much as possible the formation of agglomerates. The rest of the components were incorporated in the order indicated in Table 1, spaced every 10 min.

### 2.5. Physico-Chemical, Rheological, and Filtering Properties

The physico-chemical, rheological, and filtering properties of the different fluids were analyzed. The density (ρ, g/mL) was determined with a mud balance by weighing a specific fluid volume. The pH was determined with a potentiometer, previously calibrated with buffer solutions at pH 4 and 10. The followed procedures for rheological and filtering properties are described below.

#### 2.5.1. Rheological Properties

The rheological properties were determined with a Fann 35A concentric cylinder rotational viscometer, which measures flow resistance at six different rotational rates. First, the fluid was placed into the viscometer beaker and stirred slowly. Then, the readings were taken at the different shear rates. The plastic viscosity (µ_p_), apparent viscosity (µ_a_), and the yield point (Y_p_) were calculated as follows:

(5) $$\mu_{p}\left(\mathrm{mPa}\cdot s\right){=\theta }_{600}{-\theta }_{300}$$

(6) $$\mu_{p}\left(\mathrm{mPa}\cdot s\right){=\theta }_{600}{-\theta }_{300}$$

(7) $$Y_{p}\left(\mathrm{Pa}\right)=0.511\left(\theta_{300}{-\mu }_{p}\right)\;$$

where θ_600_ and θ_300_ are the viscometer dial readings (mPa·s) at the rotational speed of 600 and 300 rpm, respectively [21].

The gel strength, R_g_ (Pa), is the viscometer read after the fluid is maintained during 10 s and 10 min at 3 rpm.

#### 2.5.2. Filtering Properties

The filtering properties were determined according to the American Petroleum Institute (API) Standard 13 B-1 for water-based drilling fluids using the API filter press [22]. This test consists of determining the volume of filtrate collected in a given time and the analysis of the solid that is generated. It was performed at room temperature (25 °C) and a pressure of 690 kPa. The results were recorded as lost volume (mL) in 30 min (V_API_).

### 2.6. Temperature and Contaminant Resistance

The effect of temperature and pollutants on their properties of WBDFs was analyzed using three special tests: (1) High-pressure and high-temperature filtering (HPHT), (2) aging in a dynamic oven, and (3) contamination with cement and salt.

#### 2.6.1. HPHT Filtering

This test follows the same API filtering principle; however, it was performed at 147 °C and applying a differential pressure of 3.45 MPa. The filtrate was recorded as the double of the lost volume (mL) in 30 min (V_HPHT_).

#### 2.6.2. Aging in a Dynamic Oven

In this test, the fluids were exposed to high pressure (4.14 MPa) and high temperature (95 °C) for 16 h in a dynamic oven to simulate the conditions found in the draw-well. Once the fluid was aged, its rheological and filtering properties were again evaluated.

#### 2.6.3. Salt and Cement Contamination

Each WBDF was contaminated with two mixtures: One with 57.1 g/L of NaCl and the other with 11.43 g/L of cement. Their physical-chemical, rheological, and filtering properties were determined before and after they were contaminated.

## 3. Results

### 3.1. Starch Characterization

#### Content of Aldehyde and Carboxyl Groups

Ammonium persulfate (APS) is an efficient initiator for starch graft copolymerization in an aqueous medium, is inexpensive, and soluble in water [23]. In this work, the APS was successfully used for grafting an itaconic acid onto the starch sample. Table 2 gathers the content of aldehyde and carboxyl groups in the different starches. The increase of the –COOH content in S-*g*-IA_APS is indicative of the successful IA grafting on NCS [15]. Moreover, the decrease of aldehyde groups also indicates the grafting. The levels of functional groups found in CPS, suggest that this is a modified starch with carboxylic acid groups.

Figure S1 of Supporting Materials compares the FTIR spectra of NCS, S-*g*-IA_APS, and CPS. The characteristic maximum absorption bands of starch are present in the spectrum of NCS [24,25]. In the S-*g*-IA_APS spectrum, all the maximum absorption bands of NCS are observed, at approximately the same wavenumbers. Moreover, a new maximum absorption band is distinguished at 1709 cm^−1^, typical of the −C=O of aldehydes and unconjugated carboxylic acids, thus confirming the IA grafted on NCS. This is in good agreement with what has been found in the literature [17,26,27]. In the CPS spectrum, the maximum absorption band is found at 1634 cm^−1^ as a wider band with a small shoulder at 1720 cm^−1^, which is in accordance to the content of –COOH found in this product. Thus, this confirms that CPS is a modified starch with carboxylic groups.

The thermal behavior of each starch, NCS, S-*g*-IA_APS, and CPS, is presented in Figure S2 (see SI). Both NCS and CPS presented two stages of mass loss, while S-*g*-IA_APS exhibited three stages. The thermometric parameters of the different stages as well as the corresponding activation energies are included in Table S1 of Supplementary Materials. When NCS is heated under an N_2_ atmosphere, the sample did not change until approximately 250 °C, except for the loss of humidity [28,29]. In the initial stage, the loss of mass corresponds to the evaporation of free water adsorbed from the environment and of the water bounded to the hydrophilic polymer [30]. The next step is due to the main process of degradation, the depolymerization. In this process, ether bonds and unsaturated structures are formed by a thermal condensation between the hydroxyl groups of the chains, with the removal of water and other small molecules, as well as by cleavage of the hydroxyl groups in the AGU ring. Solid degradation products include esters, acids, aldehydes, and primary alcohols. Volatile products mainly include CO, CO_2_, C_2_H_4_, H_2_O, CH_2_O, and aromatic compounds [30].

The thermal degradation of the S-*g*-IA_APS copolymer presents a slightly higher water content than that of NCS, due to the presence of a greater number of –COOH groups. From the point of view of temperature, the stability of the polysaccharide decreased with the grafting of IA onto its hydrocarbon skeleton. This can be attributed to the low thermal stability of poly(itaconic acid) (PIA), as a result of the IA decarboxylation reaction along with a chain cleavage [31]. This behavior has also been described for the IA grafted to other polymers [15,31,32] and can be ascribed to the decrease in the length of the starch chains in the copolymer, due to the simultaneous hydrolysis caused by the (NH_4_)_2_S_2_O_8_/NaHSO_3_ pair. During the degradation of the PIA, a series of reactions occur between different types of carboxylic groups existing in the polymer skeleton, which could be associated with the CO formation and decarboxylation. The presence of chemical structures resulting from the rupture of the grafted PIA chains may lead to a degradation process [33].

The commercial starch, PS, presented a lower moisture content than NCS and S-*g*-IA_APS, in spite of its high –COOH content and showed a higher stability than NCS, possibly due to the presence of acid groups in its structure.

From the values of E_a_, it is easily observed that the decomposition of grafted starch proceeds rapidly with a low energy, while the degradation of NCS required a high energy and is slow. This could be due to the differences in the carboxyl groups [30,34].

**Figure 1:**
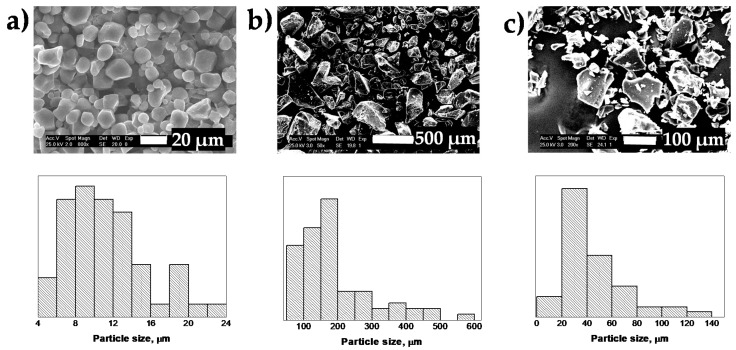
SEM micrographs (up) and particle size distribution (down) of: (**a**) Native corn starch (NCS), (**b**) copolymer (S-*g*-IA_APS), and (**c**) commercial starch (CPS).

Figure 1 includes the SEM micrographs and the granule size distribution of NCS, S-*g*-IA_APS, and CPS. It can be seen that NCS presents a bimodal distribution, typical of cereal starches, with medium granules of size between 10 and 20 μm (average particle size 14.2 μm) and small granules of size between 5 and 10 μm (size particle medium 7.7 μm) [35,36,37]. The granules are irregular in shape, from spherical to polygonal, with the highest proportion of the latter, characteristic of corn starch. The surface of the granules of NCS is uneven, granules with a completely smooth surface and others with small depressions or pores are observed.

In the case of modified starches, the structure of the starch granules is lost and the particles look fractured and fragmented [12,15]. S-*g*-IA_APS presents a polymodal granule size distribution, with the largest population of particles with sizes from 70 to 200 µm, while the commercial product CPS possesses a narrower granule size distribution with a high fraction of particles with sizes between 20–40 µm.

### 3.2. Physico-Chemical, Rheological, and Filtering Properties of WBDF

Four systems were prepared to explore the effectiveness of starches as filter controllers: A base sludge (F1) without the polymer and three WBDFs (F2, F3, and F4), in which only the starch used as a filter controller was varied (NCS, S-*g*-IA_APS, and CPS); then their properties were determined.

#### 3.2.1. Physico-Chemical and Rheological Properties

The physico-chemical and rheological parameters of the base fluid (F1) and the three WBDFs investigated (F2, F3, and F4) are collected in Table 3. Firstly, it is observed that the four fluids presented similar densities and were slightly lower than the theoretical one set for the formulations (1.5 g/cm^3^). However, a slight increase in the ρ of F2, F3, and F4 fluids is found with respect to that of the F1 base mud, caused by the addition of the filter control starch in each case. The highest ρ was found for fluid F2, whose formulation included the native NCS corn starch. It is crucial to achieve the required ρ or weight of the fluid, since this parameter counteracts the hydrostatic pressure in the formation. The pressure exerted by the fluid column should ideally be only slightly above than that of the formation to ensure the maximum penetration speed and prevent the formation fluids from entering the draw-well. If the ρ of the mud is not sufficient, it cannot withstand the formation pressure and, therefore, a potential disaster such as blows and perhaps even the explosion of the draw-well can occur. In contrast, if the ρ of the mud is very high, the possibility that the filtration of the drilling fluid penetrates into the formation increases, which would aggravate the problem of fluid loss [8].

The pH value, which represents the acid-base property of the drilling mud, is directly related to the degree of dispersion of the clay platelets in the drilling fluid; therefore, it affects the viscosity, shear, and other performance parameters of the mud. In practice, the pH value is controlled between 8–11 in most drilling fluids, to maintain a weak alkaline medium. In Table 3, it is observed that the amount of NaOH established in the formulations is adequate to achieve pH values in the recommended range. Firstly, an alkaline pH environment favors hydration of the clay [38]. Secondly, increasing the pH results in the gradual conversion of the –COOH groups to the ionized −COO^−^ state. This would increase the electrostatic repulsions between the macromolecules and the interactions between the clay platelets and the polymer chains [39]. Additionally, at this pH level the adverse effects of the contaminating electrolytes, the corrosion rates, and the bacterial action on the organic materials present in the mud are reduced.

The viscosity of the fluid at a certain shear rate, µ_a_, is the parameter of the Bingham plastic model; specifically, µ_p_ is the slope of the shear stress versus shear rate above the threshold of a plastic yield or yield point. Μ_p_ is generally explained as the part of the flow resistance caused by friction between solid particles [40]. It is affected by the concentration of solids, size and shape of the solid particles, and viscosity of the fluid phase. Both µ_a_ and µ_p_ are responsible for the pressure drop due to friction in the laminar regime, with a fundamental influence on the pump pressure in the draw-well and on the hydraulic friction [41].

The F1 base fluid has a µ_p_ value greater than that recommended for the bentonite sludge (5.0–9.0 mPa·s). The bentonite clay consists of overlapping layers, formed by two tetrahedral Si-O sheets that frame an octahedral sheet of Al–O–OH. Due to their crystalline structure, the minerals of the clay present two types of surfaces electrically and crystallographically different. On one hand, there are surfaces that correspond to the tetrahedral layers (basal planes) and, on the other hand, the edges have broken primary bonds in the tetrahedral and octahedral layers. The basal surfaces are negatively charged, while the edges are positive. In an aqueous dispersion, water can penetrate the interlaminar layer space and cause swelling of the bentonite. The behavior of the suspension of bentonite in water is complex, due to the non-isometric clay platelets that expose different glass faces, on which a double electrical layer can be developed, which differs both in the sign and in the magnitude of the total area potential. Consequently, the rheological characteristics in bentonite-water suspensions are explained by the clay particle-clay particle and clay particle-water interactions. Taking into account the shape and the surface charge, it is possible to form various associations between the clay particles in an aqueous suspension; that is, edge-face, edge-edge, and face-face interactions [39,42].

In the absence of electrolytes, the clay particles have their unaltered double ionic layers, formed exclusively by their exchange cations. Then, the interactions of the particles will depend only on their electric charge or surface charge density. The flocculation of bentonite by electrolytes is the result of a change in particle-particle associations, which is determined by the electrostatic repulsions between the double layers of two similar charged sheet surfaces; the attraction between adjacent platelets by van der Waals forces (face-face and edge-edge interactions), and by electrostatic forces between negatively charged surfaces and positively charged edges (face-edge interactions); and by the hydration forces caused by the absorption of water from the clay surface and the interchangeable cations [42].

The F1 base fluid contains, in addition to bentonite, BaSO_4_, NaHCO_3_, and NaOH. BaSO_4_ is an inert solid whose function is to provide weight to the mud. NaOH guarantees the pH that favors the hydration of the clay [39], in an alkaline medium decreases the volume of free water, which leads to an increase in viscosity. Furthermore, OH-ions resulting from the dissociation of NaOH tend to be selectively adsorbed onto the positive edges of the particles, causing dispersion of the suspension. When electrolytes such as NaHCO_3_ are added to a bentonite suspension, the thickness of the ionic double layer of the particles decreases, the platelets get closer, the repulsive forces decrease, and edge-face bonds are formed; all these processes contribute to increase the viscosity.

The three starches added to the sludge as filter controllers F2, F3, and F4, present a diluting effect on the bentonite suspension; that is, they caused a reduction in the viscosity of these compared to that of F1. In the drilling fluid industry, low molecular mass anionic polymers are generally used to reduce viscosity or prevent flocculation, which neutralize positive charges at the edges of the clays. Some examples of these polymers include polyphosphates, lignosulfonates, polyacrylamides, and polyacrylates [4,22,39,43,44]. The three investigated starches contain –COOH groups, which are ionized at the basic pH of the fluids, giving them an anionic character and therefore a diluting effect. It seems contradictory that the greatest diluting effect is provided by NCS, with the lowest content of –COOH; however, this starch has the highest content of –C(O)H. The –C=O groups have an electronic cloud that gives them a negative partial charge, so they can also electrostatically interact with the positively charged edges of the bentonite. Another aspect to take into account is the size of the particles. Smaller starch particles produce higher viscosities, due to the greater volume of particles in the mud, which increases the friction between the solids and prevents the flow [40]. This explains why F3, formulated with S-*g*-IA_APS, presents lower viscosities than F4, formulated with CPS. Despite the fact that the copolymer contains more –COOH, its particles are larger than those of CPS.

The Y_p_ is a measure of the attractive forces between the particles in the mud, under dynamic or flow conditions [40]. In the Bingham plastic model, the Y_p_ is the minimum shear stress that must be applied for a fluid to start flowing [41]. This parameter is related to the fluid cleaning function under dynamic conditions and is an indicator of the mud ability to transport suspended solids [41]. Drilling fluids with high Y_p_ values are required as they are more capable to transport the perforation cuts. However, if its value is extremely high, a higher power pump will be required to promote the cutting drillings to the surface [45]. The recommended Y_p_ value for bentonite fluids is 6.0−12.5 Pa [20].

The formulated fluids presented acceptable Y_p_ values, except for F1. It has been found that clay can suspend and transport cuttings due to their swelling properties. The presence of an additional dispersant in F2, F3, and F4, implies a greater power required to overcome the additional resistances, for this reason Y_p_ increases [45]. Furthermore, the high Y_p_/µ_p_ ratios of F3 and F4, indicate that these are shear thinning sludges, which are necessary for the drilling fluid to turn into a gel, which can suspend cuts when circulation stops and rapidly breakdown to a thin fluid when it is agitated by resumption of drilling [39]. The largest Y_p_ is found for F2, formulated with NCS, which also had the lowest µ_p_. Systems with Y_p_ >> µ_p_ exhibit partial solids flocculation, which can cause a loss of filtering properties, clogging of the drill string, and an overload of the pumping system [41].

The R_g_ determines the ability of the drilling fluid to suspend cuts under static conditions, and is a measure of the shear stress required to produce a sliding motion in the drilling fluid [8,41]. It has been stated that any clay/water mixture should keep the solids in suspension at an R_g_ value of 1.5 Pa [40]. In the present study, all the analyzed fluids met this minimum condition. However, for the bentonite fluids the recommended value for the difference of the initial and final R_g_ (R_g,10 min_ − R_g,10 s_) should be ranged between 2.5 to 5.0 Pa [20]. The values obtained for F3 and F4 indicate that these fluids have a tendency to form progressive gels. Fluids with progressive gelation can reach high viscosities in a short period of time and then, induce pressure points when the system circulation is restored after an operational shutdown, which could cause problems of fracture formation and loss of circulation [40,41].

In spite of the aforementioned problems, a moderate increase on the values of R_g,10 s_ and R_g,10 min_ is desirable. Drilling fluids are thixotropic in nature, that is, when they do not move, a gel structure is formed whose strength increases over time. This gel structure is necessary to allow the sludge to keep the cuts in suspension when there is no flow [46]. The viscosity of the thixotropic fluid changes over time at a constant shear rate until an equilibrium is reached. Thixotropy is necessary for rapid drilling, efficient lifting of drill cuttings, and to withstand densifying material when the mud flow stops. It is sometimes desirable for thixotropy to provide resistance to flow in order to avoid or reduce losses or flow to formation [12].

**Figure 2:**
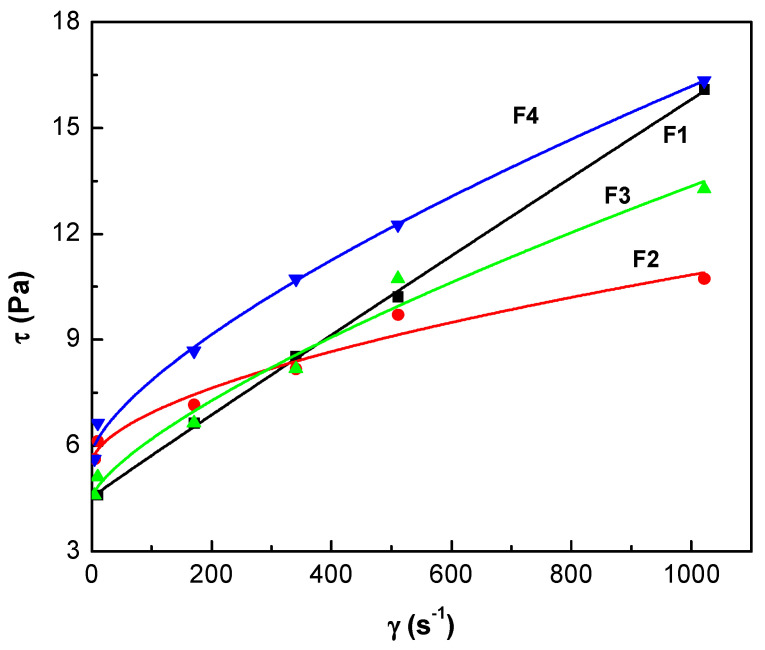
Shear stress curves as a function of shear rate for the different WBDFs. The lines represent the values predicted with the Herschel-Bulkley model.

The present formulations are complex and their properties depend on the rheology of the system. Drilling fluids exhibit a non-Newtonian behavior and the conventional rheological models widespread used in the oil industry include Bingham, Power Law, and Newton plastic models. Between them, the power law provides more information on the low shear stress condition, but still has weaknesses at high shear rates. Nowadays, the shear stress/shear rate data for many fluids place them into the elastic-pseudoplastic category, fluids that exhibit yield stress and a non-linear relationship between shear stress and shear rate once the flow starts. A three-parameter model for such fluids, proposed by Herschel and Bulkley, combines the characteristics of the Bingham plastic and power law models [47]. Three parameters characterize the Herschel and Bulkley equation for shear stress; $\tau =\tau_{0}+k\gamma^{n}$, the consistency *k*, the flow index *n*, and the yield shear stress τ_0_. The consistency is a simple constant of proportionality, while the flow index measures the degree to which the fluid is shear-thinning or shear-thickening. The experimental data and the corresponding fitting curves with this model are represented in Figure 2 and the values obtained are collected in Table S2 of Supplementary Materials.

In the interval of considered, all the samples presented a shear thinning. It is observed that the flow curve corresponding to F4 is located above the curve of F1 for all rates, while those of F2 and F3 are above only at low rates. In general, the rheology of the WBDF formulated with S-*g*-IA_APS (F3) is between that of F2 with NCS and that of F4 with CPS.

In the drilling operation, a fluid with high τ_0_ is desirable as it has a better ability to transport drill cuttings. However, since this parameter represents the minimum amount of effort that must be applied to the fluid before it starts to move, the drilling fluid with an extremely high initial value would require a high-powered pump to transport the fluid to the draw-well and to lift drill cuttings to the surface. In this study, in terms of the Herschel-Bulkley, it can be stated that all fluids are considered suitable candidates for a good drilling fluid, since their values are between 4.5 to 5.8 Pa, below the established criteria for drilling fluids (<15 Pa) [45]. The *k* parameter shows the degree of significant change in the dependent variable (τ) as a result of changes in the independent variable (γ). A higher value of *k* implies greater changes of resulting from the variation of the applied. Therefore, in the case of the studied fluids, the results indicate that in the absence or in the presence of a small amount of starch, the tension is small and is not a strong function of the applied shear rate.

Furthermore, in terms of *n* the results show that all WBDFs follow a typical pseudoplastic behavior (shear thinning), since the calculated flow indices are less than 1. According to the literature, the pseudoplastic fluid, or rather known as the shear fluid, refers to the fluid that has a lower µ_a_ at higher and high µ_a_ at low. The F1 base fluid exhibits a slightly pseudoplastic behavior, which can be attributed to the presence of bulky swollen clay structures when the fluid moves at low, which causes the clay to become entangled in the random direction resulting in a greater flow resistance [45]. With the addition of the polymers in fluids F2, F3, and F4, the pseudoplastic character is accentuated. In general, in rheological fluid studies, the pseudoplastic behavior is associated with a fluid with large molecules (commonly polymeric materials) in a solvent with smaller molecules. A typical pseudoplastic fluid shows that at lower, most macromolecular chains drop and tangle randomly, and consequently the fluid exhibit a high resistance to the flow. In contrast, at a higher, the fluid will gradually align in a direction parallel to the flow and will produce less resistance.

#### 3.2.2. Filtering Properties

**Figure 3:**
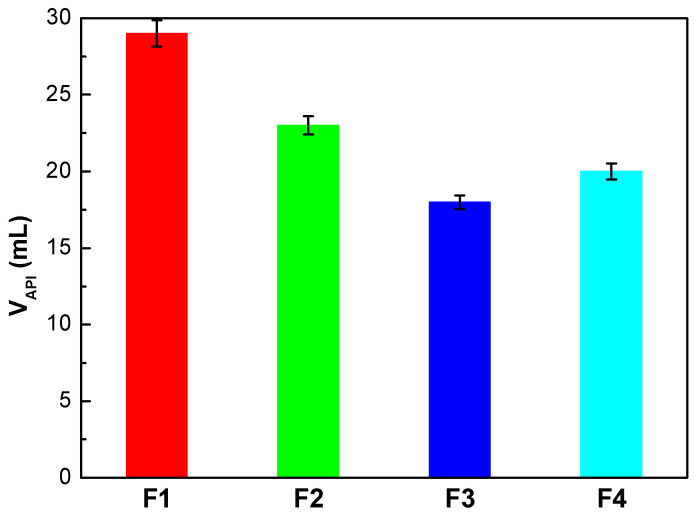
Filtering volume of drilling fluids.

**Figure 4:**
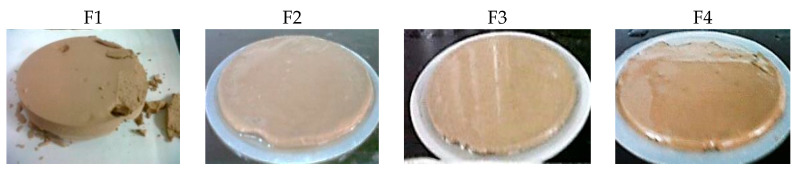
Filter cakes of the different WBDFs.

The influence of the liquid phase of the fluid (filtrate) on the rock formation must be controlled to avoid the damage into the oil and gas production areas [8,41]. Figure 3 displays the collected volume in the static filter test obtained at 25 °C and 690 kPa for the four fresh fluids. The objective of this test is to determine the behavior of the fluid in the periods when the pumping is stopped. The F2, F3, and F4 sludges present a reduction in V_API_ of 21%, 38%, and 31%, respectively, in comparison with F1. However, it was not possible to decrease the V_API_ to the recommended values (<6 mL) [20]. The introduction of charged starches in the formulation allows starches to effectively adsorb to both positive clay edges and negatively charged faces, through electrostatic attraction and hydrogen bonding [38,39]. For this reason, F3 and F4 present superior results than F2. Then, the addition of 1.5 g of starch into the drilling fluid results in a reduction of filtrate loss because the filter cake formed is thinner, more compact, and elastic compared to that of the drilling fluid without an added polymer (see Figure 4 for illustration). A thick filter cake such as in the case of F1 would produce adverse effects, such as differential pressure sticking problems [40].

### 3.3. WBDF Resistance to Temperature and Contaminants

#### 3.3.1. HPHT Filtering

Figure S4 of Supplementary Materials compares the results of V_API_ (25 °C, 690 kPa) and V_HPH_T (147 °C, 3.45 MPa) for the three WBDFs of interest. It is remarkable that with the increase in temperature and pressure, the loss of fluid increased. It has been stated that this behavior caused by the increase in temperature is expected, through three possible mechanisms: i) Reduction of the viscosity of the filtrate, ii) changes in the electrochemical balance, which governs the degree of flocculation of the suspended solids and the permeability of the filter cake, and iii) degradation of starch or other components of the fluid [41]. Although none of the systems showed V_HPHT_ values in the recommended range for WBDF (12–24 mL), it is proper to highlight that S-*g*-IA_APS improved F3 filtering at high pressure and temperature by 38%, with respect to the control made by NCS in F2. The commercial product CPS presented an improvement in filtering of 49% under the same conditions. The ability of S-*g*-IA_APS and CPS to control filtering better than NCS is due to the presence of –COOH groups. The higher efficiency of CPS compared to S-*g*-IA_APS can be attributed to its higher initial decomposition temperature and its smaller particle diameter, at high temperature the CPS particles probably swelled and absorbed more water than those of the copolymer [40]. Moreover, the porosity and permeability of the filter cake is usually affected by pressure. A useful check to determine cake compressibility is the V_HPHT_/V_API_ ratio, where the lower this ratio is, the more compressible the filter cake will be. If the compressibility ratio is greater than 1.5, it could indicate that the colloidal fraction is inadequate. In the present study, this parameter gives values of 3.4, 2.7, and 2.0 for F2 > F3 > F4, respectively.

#### 3.3.2. Thermal Aging Test

High temperatures are frequently found in drilling deep draw-wells. They have a negative effect to the control of sludge filtration, mainly due to hydrolysis, depolymerization, and other chemical degradations of drilling sludge additives, as well as flocculation or irreversible transformation of clays [4,48]. The aging test results for the different WBDFs are collected in Table S3 of Supplementary Materials. The most significant changes are the increase in µ_p_ and the decrease in Y_p_ in F2 and F3 sludges, and also the reduction of R_g,10s_ in all containing starches. High temperatures cause clay particles to flocculate easily, resulting in increased viscosity and water loss. The gelatinizing properties of starch are known to be responsible for its ability to control fluid viscosity and control fluid loss in the drilling mud [1]. The increase in the viscosity in F2 can be explained by the swelling of the granules of NCS at 95 °C without thermal degradation. In the case of F3, which contains S-*g*-IA_APS that is gelatinized on its obtaining process, the behavior could be due to the presence of −COO^−^ groups, which reduce the degree of intramolecular rotation, allowing the chains to curl more easily [12]. The reduction in Y_p_ values can be explained due to the decrease of attractive forces between the particles in the mud with the heat treatment. Despite the decrease in the initial strength of the gel, the fluids maintain the thixotropic character required to keep the solids in suspension. The slight increase in V_API_ values could be the result of the formation of a filter cake with higher permeability [5].

#### 3.3.3. Salt and Cement Contamination

Table 4 collects the physico-chemical, rheological, and filtering characteristics of fluids contaminated with salt and cement. The addition of NaCl to the sludge causes a slight decrease in density and in pH; an increase in rheological properties, markedly the µ_a_ and Y_p_ of F2 and F4; an increase in fluid loss, more pronounced in F2 and F4.

**Figure 5:**
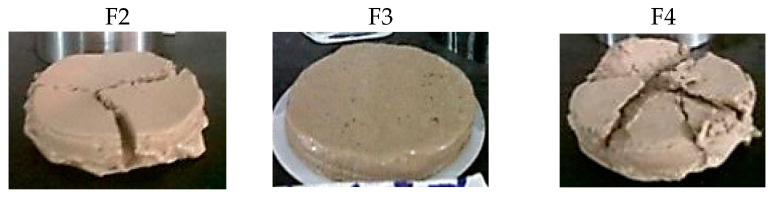
Filter cakes contaminated with salt.

Na^+^ ions tend to replace some of the H^+^ and Ca^2+^ ions on clay surfaces, slightly lowering the pH and probably increasing the soluble Ca^2+^ content. From the point of view of the filter controller, Na^+^ may shield the charges of the −COO^−^, thereby decreasing the ionic repulsions between the starch chains, contributing to the increase in V_API_ [39]. However, the results show that sludge containing S-*g*-IA_APS presents good resistance to salts. The presence of a higher amount of −COO^−^ groups in this material increases the number of charges, causing an enormous steric impediment. In addition, a thick hydration layer forms around the macromolecules and protects the counterion, improving the F3 resistance to salt [12]. The quality of the filter cakes (see Figure 5) confirms that S-*g*-IA_APS was more tolerant to the NaCl contamination. Although a thick cake is obtained for F3, it is thinner, more compact, and flexible than those produced by F2 and F4.

The cement contamination provokes an increase of pH for all three WBDFs. Cement contains tricalcium silicate (3CaO·SiO_2_) and tricalcium aluminate (3CaO·Al_2_O_3_), which react with water to form lime, Ca(OH)_2_. This compound in the solution causes most of the difficulties associated with cement contamination. Lime in drilling fluids causes chemical reactions that can be unfavorable to fluid loss and rheological properties.

The presence of hydroxyl ions increases the pH, while Ca^2+^ affects the characteristics of the clay. If the pH reaches very high levels, the chemical state of the polymers used to control the filtering is altered [49]. Both µ_a_ and µ_p_ increase with the incorporation of cement. Fresh water bentonite systems are flocculated by the cement, which will lead to increased rheology and fluid loss [49]. Ca^2+^ tends to replace Na^+^, leading to the flocculation of clay particles. The water layer bound between the clay platelets is also reduced, resulting in decreased hydration or swelling characteristics. Surprisingly, the Y_p_ values of the three WBDFs decrease significantly and, the R_g_ and V_API_ values are slightly affected. This behavior could be explained by the pretreatment of the WBDFs with NaHCO_3_, which reacts with lime. Then, H^+^ is released consuming OH^−^, which provokes a reduction of pH, allowing the dissolution of additional lime [49,50].

## 4. Conclusions

A graft copolymer (S-g-IA_APS) of an itaconic acid was synthesized from native corn starch (NCS) by conventional radical dispersion polymerization using the redox initiation system (NH_4_)_2_S_2_O_8_/NaHSO_3_. The TGA analysis showed that S-g-IA_APS was thermally more stable than NCS. Three different starches were used as filter controllers, NCS, graft copolymer (S-g-IA_APS), and commercial starch (CPS). The three starches caused a reduction in the viscosity of the bentonite suspension. The drilling fluids were thixotropic in nature, with a pseudoplastic behavior. The S-g-IA_APS improved filtering at a high pressure and temperature by 38%. The thermal ageing treatment affected the rheological properties of the three WBDFs, maintaining the thixotropic character and the fluid loss control properties. Only the S-g-IA_APS containing formulation showed a good resistance to salts. The NaHCO_3_ pretreatment allowed the cement contamination and salt contamination to resist for all WBDFs.

## Figures and Tables

**Table 1:** Products used for the formulation of the water-based drilling fluids (WBDFs).

Component	Amount (g)	Fluid	Starch	Function
Water	350			Continuous phase
Starch	1.5	F1	Without	Filter controller
		F2	NCS	
		F3	S-*g*-IA_APS	
		F4	CPS	
Bentonite	17.5			Viscosifying
BaSO_4_	219.3			Densifying
NaHCO_3_	2.0			Antimicrobial
NaOH	0.5			pH controller

**Table 2:** Aldehyde and carboxyl content of starches.

Starch	–C(O)H (%)	–COOH (%)
NCS	0.79 ± 0.03	0.05 ± 0.01
S-*g*-IA_APS	0.08 ± 0.02	0.42 ± 0.04
CPS	0.03 ± 0.02	0.31 ± 0.04

**Table 3:** The physico-chemical and rheological parameters of the different fluids.

Property	F1	F2	F3	F4
ρ (g/mL)	1.43	1.46	1.44	1.45
pH	9.2	10.2	10.4	10.1
µ_a_ (mPa·s)	15.8	10.5	13.0	16.0
µ_p_ (mPa·s)	11.5	2.0	5.0	8.0
Y_p_ (Pa)	4.3	8.7	8.2	8.2
Y_p_/µ_p_ (s^−1^)	378	4342	1635	1022
R_g,10 s_ (Pa)	3.6	5.6	4.6	7.2
R_g,10 min_ (Pa)	6.1	6.6	9.2	12.8
R_g,10 min_ − R_g,10 s_ (Pa)	2.5	1.0	4.6	5.6

**Table 4:** Rheological and filtering parameters of contaminated WBDFs.

Parameter	F2	F3	F4
Salt Contaminated WBDF			
ρ (g/mL)	1.36	1.43	1.30
pH	10.4	9.8	9.5
µ_a_ (mPa·s)	44.5	17.0	36.0
µ_p_ (mPa·s)	9.0	6.0	6.0
Y_p_ (Pa)	36.3	11.2	30.7
Y_p_/µ_p_ (s^−1^)	4030	1873	5109
R_g,10s_ (Pa)	23.5	7.7	14.8
R_g,10 min_ (Pa)	26.1	11.8	17.9
R_g,10 min_ − R_g,10 s_ (Pa)	2.6	4.1	3.1
V_API_ (mL)	154	85	173
Cement Contaminated WBDF			
ρ (g/mL)	1.46	1.45	1.45
pH	12.4	12.5	12.5
µ_a_ (mPa·s)	14.0	15.0	16.5
µ_p_ (mPa·s)	12.0	13.0	14.0
Y_p_ (Pa)	2.0	2.0	2.6
Y_p_/µ_p_ (s^−1^)	170	157	182
R_g,10 s_ (Pa)	0.5	0.5	0.5
R_g,10 min_ (Pa)	6.6	6.6	6.1
R_g,10 min_ − R_g,10 s_ (Pa)	6.1	6.1	5.6
V_API_ (mL)	26	16	13

## Supplementary Materials

The following are available online at https://www.mdpi.com/1996-1944/13/12/2794/s1, Figure S1: ATR-FTIR spectra of (a) NCS, (b) S-g-IA_APS, and (c) PS, Figure S2: Thermograms (left) and derivative (right) of (a) NCS, (b) S-g-IA_APS, and (c) PS, Figure S3: Schematic representation of the interactions between bentonite and S-g-IA_APS, Figure S4: Comparison between VAPI and VHPHT of drilling fluids, Table S1: Thermogravimetric parameters of starches, Table S2: Herschel-Bulkley parameters of WBDF, Table S3: Rheological and filtering parameters of aged WBDF.
