# Supplementary material for: Modified Starch as a Filter Controller in Water-Based Drilling Fluids
Source: Materials (Basel). 2020 Jun 20;13(12):2794. doi: 10.3390/ma13122794 (PMC7345978; doi:10.3390/ma13122794)
Supplement: Supplementary file 1 [file materials-13-02794-s001.pdf]

materials

MDPI

Supplementary Materials

Modified starch as a filter controller in water-based drilling fluids

Diana Soto [1], Orietta León [1,*], José Urdaneta [1], Alexandra Muñoz-Bonilla [2,3] and Marta Fernández-García [2,3,*]

[1] Laboratorio de Polímeros y Reacciones, Escuela de Ingeniería Química, Facultad de Ingeniería, Universidad del Zulia, Sector Grano de Oro, Maracaibo 4011, Venezuela; dsoto@fing.luz.edu.ve (D.S.); joseurdaneta1@hotmail.com (J.U.)
[2] Departamento de Química y Propiedades de Materiales Poliméricos, Instituto de Ciencia y Tecnología de Polímeros (ICTP-CSIC), C/Juan de la Cierva 3, 28006 Madrid, Spain; sbonilla@ictp.csic.es
[3] Interdisciplinary Platform for Sustainable Plastics towards a Circular Economy-Spanish National Research Council (SusPlast-CSIC), 28006 Madrid, Spain
* Correspondence: orleon@fing.luz.edu.ve (O.L.); martafg@ictp.csic.es (M.F.-G.); Tel.: +58-4246735743 (O.L.); +34-912587530 (M.F.-G.)

Received: 5 May 2020; Accepted: 18 June 2020; Published: 20 June 2020

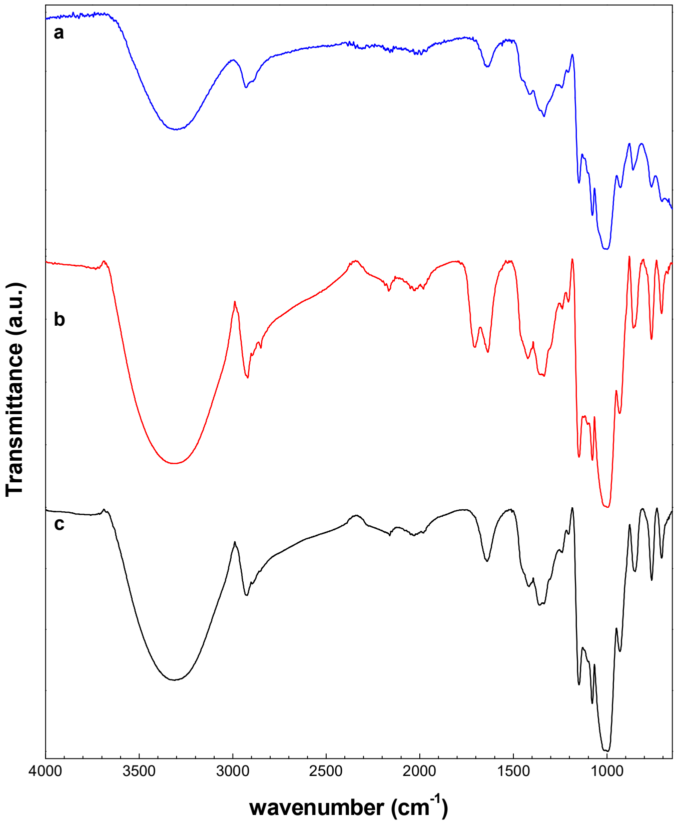

Figure S1. ATR-FTIR spectra of (**a**) NCS, (**b**) S-*g*-IA_APS, and (**c**) PS.

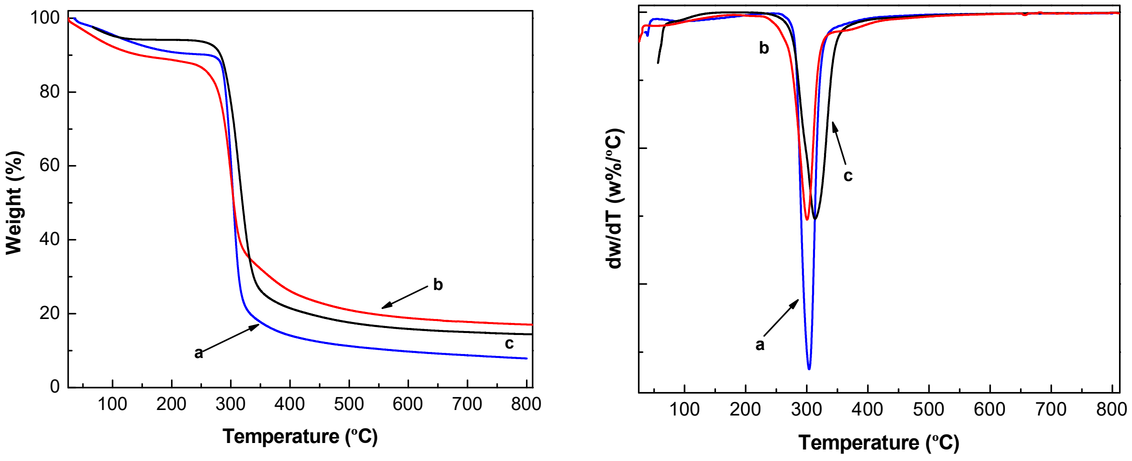

(**a**) (**b**)

Figure S2. (**a**) Thermograms and (**b**) derivative: a—NCS, b—S-*g*-IA_APS, and c—PS.

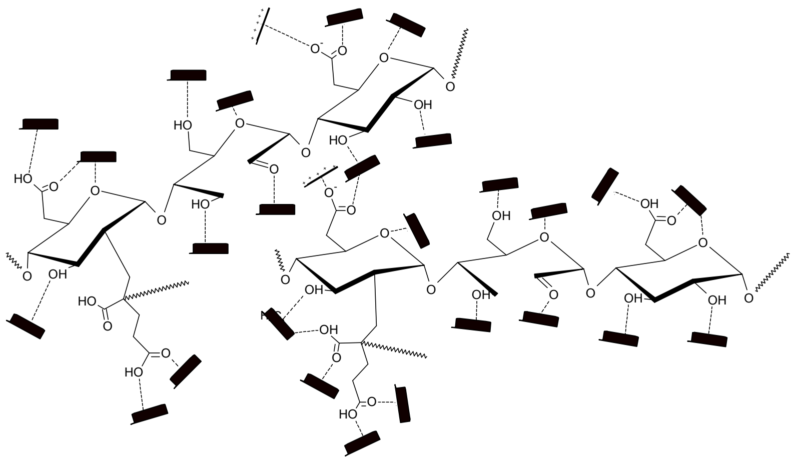

Figure S3. Schematic representation of the interactions between bentonite and S-*g*-IA_APS.

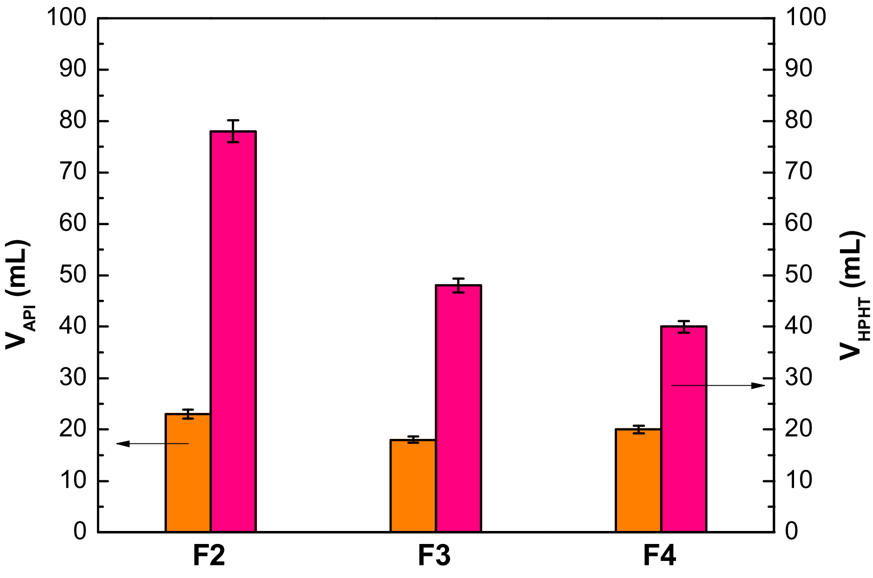

Figure S4. Comparison between V_{API} and V_{HPHT} of drilling fluids.

Table S1. Thermogravimetric parameters of starches.

Sample	Step	Temperature Range (°C)	T_{max} (°C)	Weight Loss (%)	Ash at 800 °C (%)	E_a (kJ/mol)
NCS	1	35–214	104	9.5		
	2	250–413	304	77.0	8	208
S-*g*-IA_APS	1	35–225	108	11.0		
	2	225–342	300	55.0		129
	3	342–483	413	12.0	17	18
PS	1	35–155	65	6.0		
	2	212–450	314	75.0	14	140

T_{max} is the temperature at the highest rate of mass loss.

Table S2. Herschel-Bulkley parameters of WBDF.

Parameter	F1	F2	F3	F4
τ_0 (Pa)	4.531	5.491	4.518	5.757
k (Pa·s^n)	0.013	0.105	0.060	0.084
n	0.978	0.569	0.724	0.698
R^2	0.999	0.954	0.971	0.992

Table S3. Rheological and filtering parameters of aged WBDF.

Parameter	F2	F3	F4
	Fresh WBDF		
μ_a (mPa·s)	10.5	13.0	16.0
μ_p (mPa·s)	2.0	5.0	8.0
Y_p (Pa)	8.7	8.2	8.2
Y_p/μ_p (s^{-1})	4342	1635	1022
$R_{g10\,s}$ (Pa)	5.6	4.6	7.2
$R_{g10\,min}$ (Pa)	6.6	9.2	12.8
$R_{g,10\,min} - R_{g,10\,s}$ (Pa)	1.0	4.6	5.6
V_{API} (mL)	23	18	20
	Aged WBDF		
μ_a (mPa·s)	11.0	10.0	11.5
μ_p (mPa·s)	10.0	8.0	7.0
Y_p (Pa)	2.0	4.0	9.0
Y_p/μ_p (s^{-1})	200	500	1286
$R_{g,10\,s}$ (Pa)	1.0	0.5	2.6
$R_{g,10\,min}$ (Pa)	6.1	4.6	5.1
$R_{g,10\,min} - R_{g,10\,s}$ (Pa)	5.1	4.1	2.6
V_{API} (mL)	26	20	23

© 2020 by the authors. Submitted for possible open access publication under the terms and conditions of the Creative Commons Attribution (CC BY) license (http://creativecommons.org/licenses/by/4.0/).
